# Genetic Polymorphism and Expression of CXCR4 in Breast Cancer

**DOI:** 10.1155/2015/289510

**Published:** 2015-10-20

**Authors:** Marina Okuyama Kishima, Karen Brajão de Oliveira, Carolina Batista Ariza, Carlos Eduardo Coral de Oliveira, Roberta Losi Guembarovski, Bruna Karina Banin Hirata, Felipe Campos de Almeida, Glauco Akelinghton Freire Vitiello, Kleber Paiva Trugilo, Alda Fiorina Maria Losi Guembarovski, Walter Jorge Sobrinho, Clodoaldo Zago Campos, Maria Angelica Ehara Watanabe

**Affiliations:** ^1^Laboratory of Human Pathology, Department of Pathology, Clinical Analysis and Toxicology, State University of Londrina, Londrina, PR, Brazil; ^2^Department of Pathological Sciences, Biological Sciences Center, State University of Londrina, Pr 445 Km 380 Celso Garcia Cid Highway, 86057-970 Londrina, PR, Brazil; ^3^Gineco-Med Clinical of Mastology, Londrina, PR, Brazil; ^4^Cancer Hospital of Londrina, Londrina, PR, Brazil

## Abstract

*CXCR4* genetic polymorphisms, as well as their expression level, have been associated with cancer development and prognosis. The present study aimed to investigate the influence of* CXCR4* rs2228014 polymorphism on its mRNA and protein expression in breast cancer samples. It was observed that patients presented higher* CXCR4* mRNA relative expression (5.7-fold) than normal mammary gland, but this expression was not correlated with patients clinicopathological features (nuclear grade, nodal status, ER status, PR status, p53 staining, Ki67 index, and HER-2 status). Moreover,* CXCR4* mRNA relative expression also did not differ regarding the presence or absence of T allele (*p* = 0.301). In the immunohistochemical assay, no difference was observed for CXCR4 cytoplasmic protein staining in relation to different genotypes (*p* = 0.757); however, high cytoplasmic CXCR4 staining was verified in invasive breast carcinoma (*p* < 0.01). All in all, the results from present study indicated that rs2228014 genetic variant does not alter* CXCR4* mRNA or protein expression. However, this receptor was more expressed in tumor compared to normal tissue, in both RNA and protein levels, suggesting its promising applicability in the general context of mammary carcinogenesis.

## 1. Introduction

Chemokines, identified on the basis of their ability to induce chemotaxis, have a fundamental role not only in inflammation and immune surveillance but also in cancer progression [1]. Chemokines, secreted by the tumor cells from primary tumors or metastatic sites or by the stromal cells recruited and/or locally activated, can behave as growth factors [2], increase metastasis formation and angiogenesis [3], or induce the formation of an immunosuppressive microenvironment.

Chemokine receptor 4 (CXCR4) is a transmembrane receptor that belongs to the CXC chemokine receptor family and was initially reported to mediate leukocytes homing into SDF1/CXCL12 producing tissues [4]. In addition, this receptor was reported to be expressed by cancer cells [5, 6]. Many retrospective studies have documented that the expression of various chemokine receptors, particularly CXCR4, was associated with a poor prognosis in patients with melanoma [7] and breast cancer [8].

The* CXCR4* gene is located on chromosome 2q2, in which a single nucleotide polymorphism (SNP), rs2228014 (C/T), was found at codon 138 [9, 10]. Teng et al. [11] showed that this polymorphism was associated with stages III and IV and also lymph nodes metastasis of oral cancer. Otherwise, Cacina et al. [12] have not found any significant association between CXCR4 polymorphism and endometrial carcinoma susceptibility.

Jin et al. [13] showed that the interaction between CXCL12 secreted by endothelial cells and CXCR4-expressing tumor cells is sufficient to stimulate transendothelial migration. These results suggested that CXCL12/CXCR4 axis is important in angiogenesis and tumor cell dissemination. Because both proteins were readily identifiable in a significant fraction of human breast cancer samples by immunohistochemistry, CXCR4 may constitute a molecular target for therapy.

CXCR4 may be overexpressed in breast cancer [14], and the CXCR4/CXCL12 axis is suggested to be involved in migration and consequently in the invasion and metastasis of breast cancer cells [15]. Kang et al. [16] showed that in human breast cancer tissues the level of CXCR4 expression is significantly correlated with lymph node metastasis and suggested that this receptor may be a useful prognostic indicator and a potential therapeutic target in breast cancer therapy.

In this context, the aim of this study was to investigate the influence of CXCR4 rs2228014 genetic polymorphism on its gene and protein expression in breast tumor samples.

## 2. Material and Methods

Following approval from the Human Ethics Committee of State University of Londrina (CEP/UEL 189/2013-CAAE 17123113400005231), tissue samples were collected from breast cancer patients. A term of free and informed consent was signed by all sample donors and medical doctors involved prior to biologic material collection. Clinical staging was determined according to the Union of International Control of Cancer classification criteria [17]. Samples of invasive breast carcinoma tissue were obtained from 74 female patients free of adjuvant or neoadjuvant chemotherapy, who had undergone surgery at the Cancer Hospital of Londrina, Paraná, Brazil. The tumor-node-metastasis (TNM) system was used to classify the disease status based on the major morphological attributes of malignant tumors that were thought to influence disease prognosis: size of the primary tumor (T), presence and extent of regional lymph node involvement (N), and presence of distant metastases (M).

### 2.1. DNA Extraction from Breast Tissues

Genomic DNA was obtained from tissue samples of invasive breast carcinoma by salting-out method [18] and was quantified by NanoDrop 2000c Spectrophotometer (Thermo Scientific Inc., Wilmington, USA) at a wavelength of 260 nm and 280 nm.

### 2.2. Polymerase Chain Reaction (PCR): CXCR4

DNA (100 ng) was analyzed using specific primers for* CXCR4* in a PCR reaction (GenBank accession number NM_003467.2). Primers sequences were as follows: Forward 5′-AACTTCCTATGCAAGGCAGT-3′ and Reverse 5′-TATCTGTCAT CTGCCTCACT-3′. Samples were amplified using the buffer kit plus 1.25 units of Taq polymerase (Invitrogen, Carlsbad, California, USA). PCR conditions were 5 min denaturation at 94°C, 35 cycles of 45 sec at 94°C, 1 min at 60°C and 1 min and 15 sec at 72°C, and 10 min elongation at 72°C in a Hybaid PCR Sprint Thermal Cycler (Biosystems, Guelph, Ontario, Canada). Amplicons of 236 base pairs were analyzed by electrophoresis in 2% agarose gel and visualized using UV fluorescence after staining with Blue Green reagent (LGC Biotecnologia, Sao Paulo, Brazil). All reactions were conducted with a negative control to ensure no contamination.

### 2.3. CXCR4 Genotyping

PCR products were subjected to restriction digestion by incubating with* BccI* (New England Biolabs, UK) for 4 h at 37°C. The enzymatic restriction products were analyzed by electrophoresis on 10% polyacrylamide gel and detected by a nonradioisotopic technique using silver staining. When the allelic variant is present, a change from cytosine (C) by thymine (T) at position 3952 of the initiation codon 138 eliminates the restriction site [19]. A product of 103 and 133 base pairs for C allele and a product of 236 base pairs for T allele were observed, characterizing thereby three possible genotypes: TT (homozygous for the mutant allele), CT (heterozygous), and CC (homozygous for the wild-type allele).

### 2.4. RNA Isolation and Reverse Transcriptase Reaction

Total cellular RNA was extracted using TRIzol LS reagent (Invitrogen) according to manufacturer's instructions and quantified using NanoDrop 2000c Spectrophotometer (Thermo Scientific Inc., Wilmington, USA). Reverse transcriptase reaction was performed using 500 ng of RNA, 20 units of cloned Moloney Murine Leukemia Virus Reverse Transcriptase (M-MLV RT, Invitrogen), and 4 units of Recombinant Ribonuclease Inhibitor (RNaseOUT, Invitrogen) under the following conditions: 2.5 *μ*M oligo dT, 50 mM Tris HCl pH 8.3, 75 mM KCl, 1.5 mM MgCl_2_, and 1.25 mM of dNTP, at 42°C for 60 min in a Thermal Cycler.

### 2.5. Real-Time PCR (qPCR) for CXCR4

Quantitative real-time PCR (qPCR) was performed using Platinum SYBR Green qPCR SuperMix-UDG (Invitrogen) on a Step One Real-Time PCR thermal cycler (Applied Biosystems, Foster City, USA). The primers used for amplification of CXCR4 and GAPDH are described in Table 1. The qPCR reaction was performed in 40 cycles as follows: 95°C for 30 sec, 54°C for 30 sec, and 72°C for 30 sec with detection of fluorescence at each temperature increase to confirm the specific amplification. A melting curve analysis was consistently performed at the end of the reaction to check for primer-dimer artifacts and contamination. In addition, in all experiments, appropriate negative controls containing no template were subjected to the same procedure to exclude or detect any possible contamination.

Relative mRNA expression levels of* CXCR4* were calculated according to the 2^−ΔΔCT^ method [20] and normalized by the previously characterized house-keeping gene* GAPDH*. Beside adjacent normal breast tumor RNA tissue, a commercial pool of human normal mammary gland RNA (Clontech Laboratories Inc., Mountain View, CA, USA) was also used as a nonneoplastic sample.

### 2.6. Immunohistochemical Staining

For immunohistochemical analysis, 5 *μ*m of tissue sections was obtained from breast tumors samples. Samples were heated at 56°C, deparaffinized in xylene, and rehydrated in a graded alcohol series. Antigen retrieval was performed with citrate buffer and a mouse antibody for human CXCR4 (1 : 100 dilution) (eBioscience, San Diego, CA, USA) was used. The sections were stabilized at room temperature for 30 min and washed with PBS (phosphate buffered saline) and anti-mouse/rabbit HRP secondary antibody was used as second step (Bio SB Inc., Santa Barbara, CA, USA). The diaminobenzidine (DAB) chromogen system was used (Sigma-Aldrich, USA) and counter staining was performed with Gill's hematoxylin and slide mounted in Canada balsam. The markup for CXCR4 was assessed in tumor and adjacent normal tissue. The reading was performed under a light microscope (Eclipse-E200, Nikon, Japan) by qualified pathologists. The protocol for analysis of this marker was established at the research laboratory.

We adopted the German semiquantitative scoring system, considering the IHC staining intensity and area extent, which has been widely accepted and used in previous studies [21, 22]. Every lesion was given a score according to the intensity of the staining: weak staining = 1, moderate staining = 2, and strong staining = 3. Controls were performed to verify the specificity of primary antibody and all analyses were independently made by at least two pathologists. However, if there was a discrepancy in individual scores, both pathologists reevaluated the immunohistochemical sections by reaching a consensus agreement before combining the individual scores.

### 2.7. Statistical Analysis

One sample *t*-test was performed to analyze relative mRNA CXCR4 expression, using GraphPad Prism version 6.00 for Windows (GraphPad Software, La Jolla, California USA). Spearman correlation and Chi square statistical tests were used to analyze mRNA expression, protein expression, and rs2228014 polymorphism in relation to breast cancer clinical outcome, using SPSS Statistics 22.0 software (SPSS Inc., Chicago, Illinois, USA). A *p* value < 0.05 was considered statistically significant.

## 3. Results

In the present study, CXCR4 rs2228014 (C/T) genetic polymorphism and mRNA expression were assessed in 74 female breast cancer patients. The median age of the patients was 58 years (ranging from 31 to 86 years old), diagnosed at the Londrina Cancer Hospital, Parana, Brazil.

The majority of patients (90.7%, 68/74) were diagnosed with invasive ductal carcinoma, according to the clinical criteria determined by the Union of International Control of Cancer [17]. The mean of tumor size was 2.7 cm ± 1.6 cm and the median size was 2.2 cm.

**Figure 1:**
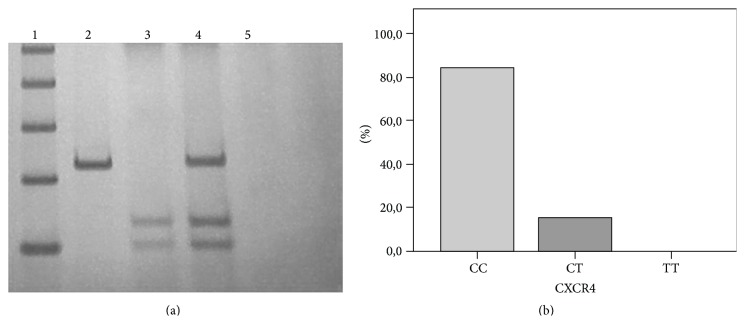
CXCR4 rs2228014 (C/T) genetic polymorphism. (a) Electrophoretic profile of rs2228014 (C/T).* BccI* restriction enzyme was used for 4 h at 37°C. Polyacrylamide gel 10% stained with silver nitrate. Lane 1, Ladder DNA fragment marker of 100 bp; Lane 2, PCR product of 236 pb; Lane 3, wild-type homozygous genotype of 133 pb and 103 pb (CC); Lane 4, heterozygous genotype of 236 pb, 133 pb, and 103 pb (CT); Lane 5, blank reaction or negative control (reaction without DNA). (b) Genotype distribution for* CXCR4* rs2228014 in breast cancer patients.

For genetic polymorphism assay, a PCR-RFLP methodology was performed, using the* BccI* restriction enzyme to examine* CXCR4* rs2228014 genotypes (Figure 1(a)). The obtained frequencies demonstrate that 58 (84.1%) of patients presented CC genotype, 11 (15.9%) of patients presented CT genotype, and there were no TT homozygotes (0.0%) (Figure 1(b)). The genotype distribution in our sample did not differ from the theoretical distribution given by the Hardy-Weinberg equilibrium (HWE).

No significant difference in* CXCR4* genotype distribution was observed according to clinicopathological features analyzed such as tumor histology (*p* = 0.686), nuclear grade (*p* = 0.312), nodal status (*p* = 0.697), estrogen receptor status (*p* = 0.630), progesterone receptor status (*p* = 0.287), p53 (*p* = 0.789), Ki67 (*p* = 0.129), and HER-2 status (*p* = 0.818) (Table 2).

**Figure 2:**
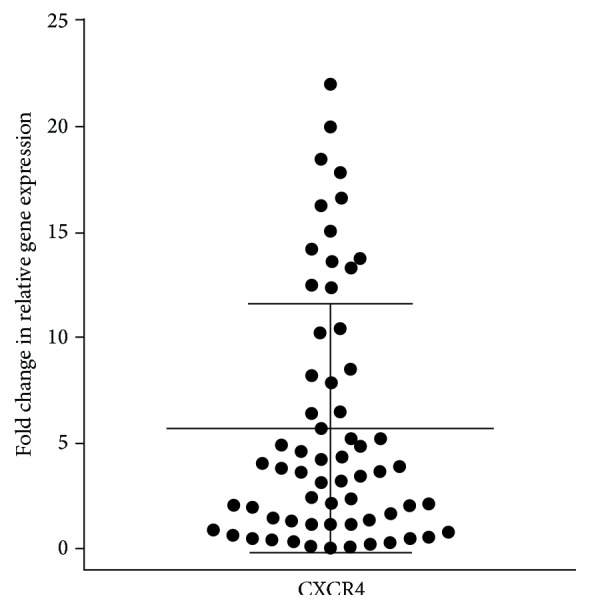
CXCR4 gene expression in tumor samples. Relative gene expression was performed by quantitative PCR using 2^−ΔΔCT^ method, in relation to mRNA from tumor-adjacent tissue and to normal mammary gland. Mean fold change = 5.7 (*p* < 0.0001).

The expression of* CXCR4* mRNA was investigated by qPCR in breast tumor tissue and in normal mammary gland. It was observed that breast cancer patients presented a higher* CXCR4* mRNA relative expression (5.7 fold) than the mRNA from normal mammary gland (Figure 2).

CXCR4 mRNA relative expression was also assessed according to clinicopathological features, such as nuclear grade (*p* = 0.549; rho = 0.079), nodal status (*p* = 0.220; rho = −0.161), ER status (*p* = 0.745; rho = 0.042), PR status (*p* = 0.189; rho = 0.169), p53 (*p* = 0.937; rho = 0.011), Ki67 (*p* = 0.810; rho = −0.034), and HER-2 status (*p* = 0.574; rho = 0.073); however, no statistical differences were observed.

**Figure 3:**
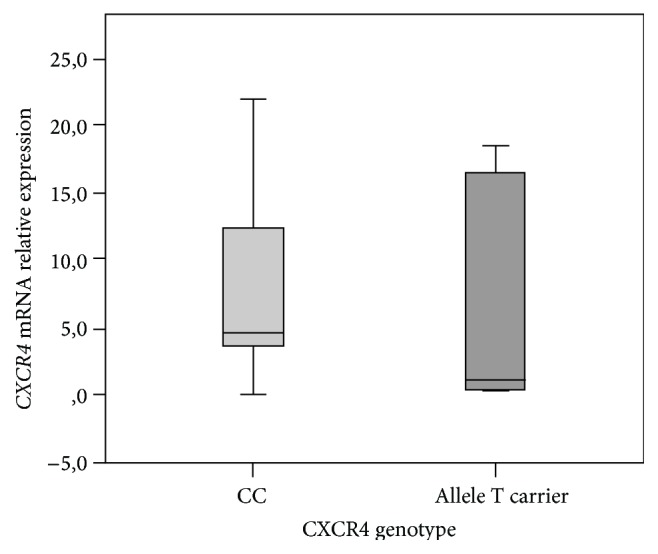
CXCR4 mRNA relative expression in accordance with rs2228014 genetic polymorphism. The Mann-Whitney test demonstrated that CXCR4 mRNA levels did not differ significantly between CC patients (mean 7.7 ± SE 5.65) and allele T carriers (mean 6.4 ± SE 8.64) (*p* = 0.301). Error bars as 95% IC.

CXCR4 mRNA relative expression was assessed in relation to rs2228014 genotypes, and the Mann-Whitney test showed no significant differences according to the presence or absence of T variant allele (*p* = 0.301) (Figure 3).

**Figure 4:**
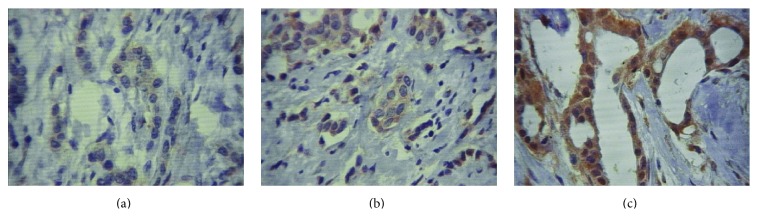
CXCR4 protein expression in breast tumor tissue samples. CXCR4 immunoreactivity was observed in the cytoplasm of tumor epithelial cells. Representative micrograph result for positive CXCR4 staining: (a) weak staining = 1, (b) moderate staining = 2, and (c) strong staining = 3. CXCR4 cytoplasmic expression in invasive breast carcinoma (400x).

In immunohistochemical assay, although no difference was observed for CXCR4 cytoplasmic protein levels compared to rs2228014 genotypes (*p* = 0.757), a high cytoplasmic CXCR4 staining was verified in invasive breast samples (Figure 4).

When CXCR4 cytoplasmic expression was verified according to breast cancer nodal status, no significant correlation was observed (*p* = 0.100; rho = −0.282). In addition, although CXCR4 protein expression did not change according to rs2228014 genotype distribution (*p* = 0.757) (Table 3), higher protein expression in the tumor microenvironment compared with adjacent normal breast tissue (*p* = 0.01) was verified.

**Figure 5:**
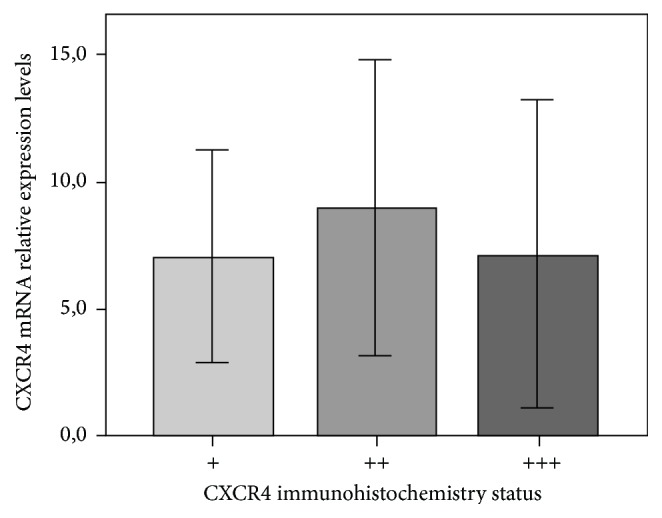
CXCR4 mRNA relative expression and immunohistochemistry status. Variance analysis tests (ANOVA) demonstrated that CXCR4 mRNA levels did not differ significantly between +/weak (mean 7.08 ± SD 6.2), ++/moderate (8.99 ± 7.0), and +++/strong (7.18 ± 7.3) (*p* = 0.809) immunohistochemistry statuses. Error bars as 95% IC.

*CXCR4* mRNA level was assessed according to its immunohistochemistry protein expression but no significant differences were observed (*p* = 0.809) (Figure 5).

## 4. Discussion

Multiple clinical, pathological, and histological features are associated with breast cancer. Fortunately, clinicopathological parameters have been validated and serve as a guide for systemic therapy and prognostication of breast cancer. These include tumor size, lymph node involvement, histological type, and grade and patients' age [23]. Moreover, estrogen is a growth factor that stimulates cell proliferation, and estrogen receptors (ER) mediate its effects [24]. Approximately 70% of breast cancers express the ER alpha and are hormone-dependent [25]. In accordance with this frequency, 67.1% of our samples expressed estrogen and progesterone receptors. Regarding the histological classification, 90.0% of patients presented invasive ductal carcinoma (IDC), which is in agreement with Harris and Solin [26], who observed 47–79% incidence in IDC and 2–15% invasive lobular carcinoma (ILC) in patients with breast cancer.

In addition, we investigated the effects of* CXCR4* gene polymorphism on the breast cancer clinicopathological development. The analysis demonstrated that 58 (84.1%) patients presented CC genotype and 11 (15.9%) the CT genotype. No significant difference in CXCR4 genotype distribution was observed according to clinicopathological features.

Kucukgergin et al. [27] have reported that* CXCR4* polymorphisms may contribute to the muscle invasive breast cancer in a Turkish population. Furthermore, Lee et al. [28] have verified that lung cancer patients carrying homozygous TT genotype of rs2228014* CXCR4* polymorphism had a tendency to develop advanced disease and poorer prognosis compared to different genotypes. Homozygous TT and heterozygous CT genotypes were also significantly associated with higher risk for renal cell carcinoma development [29].

In this work, CXCR4 genetic expression and protein detection were evaluated by real-time PCR and immunohistochemistry, respectively. It was observed that the majority of the breast cancer patients presented higher CXCR4 mRNA relative expression (5.7 fold) than mRNA from normal mammary gland and higher CXCR4 protein expression in the tumor microenvironment compared with tumor adjacent tissue. However, CXCR4 mRNA mean levels did not differ from CXCR4 immunohistochemistry status.

In this context, it has been reported that basal-like and HER2 enriched breast cancer subtypes express CXCR4 staining more often than the other subtypes. Additionally, there is also a positive relationship between lymph node involvement and CXCR4 staining of these subtypes [30]. Moreover, it is known that axillary lymph nodes positivity has been an important component for diagnosis, treatment, and subsequent research of breast cancer. Hiller et al. [31] have analyzed the literature regarding the CXCR4 expression in breast cancer metastasis to the lymph nodes and the prognostic and/or predictive implications of lymph node metastasis in the presence of elevated CXCR4. They concluded that CXCR4 level is a predictive marker for patients with locally advanced breast cancer.

Our results showed a higher cytoplasmic CXCR4 expression staining in invasive breast carcinoma tissues through the immunohistochemical assay, although it did not differ among CXCR4 genotypes (*p* = 0.757).

There is compelling evidence indicating that a subset of cancer cells, referred to as cancer stem cells, plays a critical role in tumor initiation, metastatic colonization, and resistance to therapy. Although the signals generated by the metastatic niche that regulates cancer stem cells are not fully understood, accumulating evidence suggests a key role of the CXCL12/CXCR4 axis. Cojoc et al. [32] pointed the potential for targeting the CXCL12/CXCR4 signaling pathway in cancer management, focusing on the physiological functions of this pathway in cancer and cancer stem cells.

In this context, Sobolik et al. [33] demonstrated by intravital imaging of MCF-7 cells expressing CXCR4 that tumor cells migrate toward blood vessels and metastasize to lymph nodes. Thus, CXCR4 can drive epithelial to mesenchymal transition along with an upregulation of chemokine receptors and cytokines important in cell migration, lymphatic invasion, and tumor metastasis.

Although this study did not determine distant metastasis and relapses after treatment, considerable knowledge regarding CXCR4 role in breast cancer metastasis to CXCL12 producing organs has emerged [33–36]. In view of this function, it is reasonable to assume that the evaluation of CXCR4 expression, either at mRNA or at protein levels, could be useful as an indicator of a higher risk for metastasis. Moreover, CXCR4 should be considered for the identification of patients who are likely to develop or to prevent distant metastasis. In this regard, assessing the CXCR4 expression as a molecular breast cancer biomarker is highly demanded, and this may be performed with a standardized scoring system.

Finally, this work showed increased CXCR4 expression in breast tumor tissues, at both mRNA and protein levels, but this increase is not influenced by rs2228014 genetic polymorphism. All in all, taken together, the results from present study suggest CXCR4 receptor as a promising marker in the general context of mammary carcinogenesis.

## Figures and Tables

**Table 1:** Quantitative RT-PCR conditions and primers sequences.

Gene	GenBank accession	Primer	Sequence	Melting
	number			(*T*°C)
CXCR4	AF025375	*Forward*	5′ TGTTGGCTGAAAAGGTGGTC 3′	80.5
		*Reverse*	5′ AAAGATGAAGTCGGGAATAGTC 3′	

GAPDH	NM_002046	*Forward*	5′ GAAGGTGAAGGTCGGA 3′	80.5
		*Reverse*	5′ GGGTCATTGATGGCAAC 3′	

**Table 2:** Clinicopathological parameters analysis according to rs2228014 CXCR4 genetic polymorphism in breast cancer patients.

		Total *N* (%)	CXCR4 genotype		*p* value
			CC	Allele T carrier	
			*N* (%)	*N* (%)	
Tumor histology^a^	IDC	45 (90.0)	40 (80.0)	05 (10.0)	0.686
	ILC	02 (4.0)	01 (2.0)	01 (2.0)	
	Others	03 (6.0)	03 (6.0)	00 (0.0)	

Nuclear grade	I	11 (22.0)	08 (17.4)	03 (6.5)	0.312
	II	16 (34.7)	14 (30.4)	02 (4.3)	
	III	19 (41.3)	19 (41.3)	00 (0.0)	

Nodal status	Negative	27 (60.0)	22 (48.9)	05 (11.1)	0.697
	Positive	18 (40.0)	17 (37.8)	01 (2.2)	

ER status	Negative	06 (12.5)	06 (12.5)	00 (0.0)	0.630
	Positive	42 (87.5)	36 (75.0)	06 (12.5)	

PR status	Negative	10 (20.8)	10 (20.8)	00 (0.0)	0.287
	Positive	38 (79.2)	32 (66.7)	06 (12.5)	

p53	Negative	33 (75.0)	27 (61.4)	06 (13.6)	0.789
	Positive	11 (25.0)	11 (25.0)	00 (0.0)	

Ki67	Low	17 (44.8)	12 (31.6)	05 (13.2)	0.129
	Moderate	05 (13.1)	04 (10.5)	01 (2.6)	
	High	18 (42.1)	16 (42.1)	00 (0.0)	

HER2	Negative	32 (71.1)	27 (60.0)	05 (11.1)	0.818
	Positive	13 (28.9)	12 (26.7)	01 (2.2)	

^a^IDC: Invasive Ductal Carcinoma; ILC: Invasive Lobular Carcinoma; others: CMI, ductal carcinoma in situ.

**Table 3:** CXCR4 protein expression according to rs2228014 genotypes.

		*CXCR4* genotype	
		CC *N* (%)	Allele T carrier *N* (%)
CXCR4 expression	+	09 (28.1)	02 (6.3)
	++	08 (25.0)	02 (6.3)
	+++	10 (31.2)	01 (3.1)

Pearson qui-square test; *p* = 0.757. +: weak, ++: moderate, and +++: strong.
